# The Dopamine Augmenter L-DOPA Does Not Affect Positive Mood in Healthy Human Volunteers

**DOI:** 10.1371/journal.pone.0028370

**Published:** 2012-01-04

**Authors:** John Liggins, Robert O. Pihl, Chawki Benkelfat, Marco Leyton

**Affiliations:** Department of Psychiatry, McGill University, Montréal, Québec, Canada; Centre for Addiction and Mental Health, Canada

## Abstract

Dopamine neurotransmission influences approach toward rewards and reward-related cues. The best cited interpretation of this effect proposes that dopamine mediates the pleasure that commonly accompanies reward. This hypothesis has received support in some animal models and a few studies in humans. However, direct assessments of the effect of transiently increasing dopamine neurotransmission have been largely limited to the use of psychostimulant drugs, which elevate brain levels of multiple neurotransmitters in addition to dopamine. In the present study we tested the effect of more selectively elevating dopamine neurotransmission, as produced by administration of the immediate dopamine precursor, L-DOPA (0, 100/25, 200/50 mg, Sinemet), in healthy human volunteers. Neither dose altered positive mood. The results suggest that dopamine neurotransmission does not directly influence positive mood in humans.

## Introduction

Mesolimbic dopamine (DA) neurotransmission influences the ability of rewards to elicit focused interest and approach [1]–[5]. One early and still frequently cited interpretation is that the transmitter mediates pleasure [6]. This possibility was first suggested following observations that neuroleptic medications decreased amphetamine-induced subjective “high” in stimulant drug abusers [7]–[9] and produced a sense of “psychic indifference” in patients with schizophrenia [10] while extended treatment with high doses of L-DOPA led to hypomanic states in patients with bipolar mood disorders [11]. Subsequently, a series of carefully controlled and influential animal studies indicated that increases in DA neurotransmission augmented instrumental responding for electrical stimulation of the brain (ESB) [12] while decreased DA neurotransmission disrupted responding for drugs, food, and ESB [13]–[18]. The latter effects were not attributable to compromised motor function since low doses of DA receptor antagonists increased instrumental responding while higher doses produced biphasic increases and decreases. These observations led to the suggestion that DA receptor antagonists reduced the ability to experience pleasure [6].

Some recent work is at least consistent with the anhedonia hypothesis. For example, individual differences in the magnitude of drug-induced striatal DA responses correlate with approach-related personality traits [19]–[22] and the substance's positive subjective effects [23]–[31]. In the converse experiments, mood-lowering effects of antipsychotic medications are predicted by their extent of DA D2 receptor blockade [32]–[34].

Other work, though, has seemed inconsistent with a role of DA in pleasure. First, in both humans [35], [36] and in laboratory animals [3], [37] DA release in the ventral striatum can also be evoked by aversive stimuli. Second, in operant conditioning paradigms, DA release increases and then peaks just prior to a lever press for reward and then gradually decreases thereafter [38], [39]. With experience DA comes to be released in response to cues associated with the reward [38]–[40] but not when actually receiving the reward [40], [41]. Third, an extensive series of studies has indicated that neither DA antagonists nor DA lesions alter responses in the ‘taste reactivity’ paradigm, an animal model of eating-related pleasure [2], [42], [43]. Finally, the majority of studies have failed to replicate an ability of neuroleptic medications or other DA lowering manipulations to decrease drug-induced pleasure in humans [44]–[58].

Given the above controversies, the present study aimed to test the effect of a more selective DA augmenter, L-DOPA, on mood states in healthy human volunteers. Since individual differences in approach-related traits predict differences in DA reactivity, it was further hypothesized that those who scored higher on these traits would exhibit greater mood elevation.

## Results

There were no significant Group x Time interaction effects for any of the POMS subscales (all ps>0.05, see Table 1), nor were there significant main effects of personality (all ps>0.05). A three-way Group x Personality subgroup x Time interaction raised the possibility that NS2 predicted differential POMS Agreeable-Hostile responses to L-DOPA, but this effect was no longer significant when VAS “Nauseous” scores were entered as covariates (F_6, 114_ = 0.804, p>0.05). Effects on nausea were mild (peak change = 1.4/10), and statistically significant for the 200 mg L-DOPA dose only (F_6, 126_ = 2.839, p<0.05) (see Table 1).

**Table 1:** Effect of L-DOPA on mood and nausea.

POMS Item	Group	Baseline	T2	T3	T4	Peak Change
			(+45 mins.)	(+105 mins.)	(+165 mins.)	
Composed-Anxious	Placebo	60.1±1.9	61.6±1.7	60.3±1.6	60.1±1.8	− 1.0±2.4
	100 mg L-DOPA	57.4±2.6	58.9±2.3	57.7±2.4	58.1±2.2	0.19±2.8
	200 mg L-DOPA	56.7±2.0	58.6±2.0	55.8±2.1	57.3±2.2	0.63±2.0
Elated-Depressed	Placebo	54.7±1.4	54.2±1.4	58.1±1.7	55.1±1.6	2.1±1.7
	100 mg L-DOPA	54.0±1.8	53.8±1.4	54.1±2.0	52.0±1.9	− 0.75±1.2
	200 mg L-DOPA	55.8±1.9	53.7±1.5	56.6±1.6	53.8±1.7	− 2.0±1.6
Energetic-Tired	Placebo	51.6±1.4	49.4±1.6	56.1±1.8	53.3±1.4	0.63±2.6
	100 mg L-DOPA	55.1±2.0	51.0±2.1	53.6±2.4	52.0±2.5	− 5.0±2.2
	200 mg L-DOPA	54.2±1.7	47.8±1.9	51.6±2.0	49.9±1.9	− 6.7±1.8
Agreeable-Hostile	Placebo	55.6±1.8	54.7±1.9	56.3±1.9	53.8±1.4	− 2.3±1.8
	100 mg L-DOPA	53.3±2.4	53.2±2.2	54.2±2.6	53.7±2.4	0.0±1.4
	200 mg L-DOPA	52.1±1.5	50.6±1.7	51.4±1.6	50.1±1.9	− 2.5±1.6
Confident-Unsure	Placebo	55.2±1.3	54.2±1.6	56.7±1.5	54.8±1.5	− 0.69±1.2
	100 mg L-DOPA	55.8±1.5	54.4±1.6	54.8±1.8	54.6±1.7	− 1.9±1.5
	200 mg L-DOPA	53.6±1.7	52.2±1.8	53.0±2.1	52.9±1.7	− 0.75±1.4
Clearheaded-Confused	Placebo	57.0±1.7	55.4±1.6	58.4±1.6	56.4±1.6	− 1.3±2.4
	100 mg L-DOPA	56.6±1.7	54.8±1.7	55.6±1.8	55.1±2.0	− 0.81±2.3
	200 mg L-DOPA	55.0±1.7	52.4±2.5	53.4±2.2	52.7±2.3	− 3.3±1.3
**VAS Item**						
Nauseous	Placebo	1.8±0.23	2.2±0.37	1.4±0.16	1.4±0.15	0.25±0.47
	100 mg L-DOPA	1.6±0.22	1.4±0.22	1.3±0.17	1.4±0.31	− 0.13±0.36
	200 mg L-DOPA	1.5±0.18	2.0±0.27	1.9±0.39	2.6±0.54 ^A, B, C^	1.4±0.52

(Means±SEM).

A = significantly different from placebo, B = significantly different from baseline, p<0.05, C = significantly different from 100 mg L-DOPA group at T4.

## Discussion

In the present study, the immediate DA precursor, L-DOPA, did not affect positive subjective states in healthy human volunteers, neither in the groups as a whole nor in subgroups based on hypothesized DA-related personality traits. These findings extend the results from previous drug challenge studies. In contrast to non-specific DA augmenters, such as psychostimulant drugs, which reliably and potently elevate mood in healthy human volunteers [31], [53], [59]–[61], accumulating evidence indicates that more selective DA receptor agonists do not (Table 2).

**Table 2:** The effect of dopamine-enhancing agents on positive mood states in healthy humans.

Drug	Mechanism of	Dose	Study	n	Mood	Effect on	Details
	Action				Measures	Positive Mood	
Apomorphine	Mixed D1/D2	10 µg/kg, s.c.	[95]	9	VAS	0	
	agonist						
Bromocriptine	D2 agonist	1.25 mg, p.o.	[96]	32	VAS	NR	VAS items corresponding to motivation and energy
		2.5 mg, p.o.	[97]	12	VAS	0	
		2.5 mg, p.o.	[98]	21	VAS	0	
		1.25 mg, p.o.	[99]	22	VAS	0	
		2.5 mg, p.o.	[100]	40	AMS	0	
		1.25 mg, p.o.	[101]	20	VAS	↓	Bromocriptine ↓ VAS Contented and ↑ VAS Sad and
							Antagonistic scores
		1.25 mg, p.o.	[102]	12	VAS	0	
		2.5 mg, p.o.	[103]	16	AMS	0	Not clear what these scales measure
					STAI		
L-DOPA	Selective DA	100 mg, p.o.	[97]	12	VAS	0	
	augmenter	150 mg, p.o.	[88]	14	VAS	0	
		200 mg, p.o.	[104]	22	VAS	NR	Only measured VAS “Drowsiness”
Lisuride	D2 agonist	0.2 mg, p.o.	[105]	12	VAS	↓	Adverse effects, such as nausea, vomiting and headache
							No sedative effect
Pergolide	Mixed D1/D2	0.1 mg, p.o.	[106]	40	PANAS	0	Drugs administered daily for 5 days
	agonist						No acute drug effect (assessed on day 1)
		0.1 mg, p.o.	[100]	40	AMS	0	
		0.05 mg, p.o.	[107]	15	VAS	0	
		0.1 mg, p.o.	[103]	16	AMS	↓	Not clear what these scales measure
					STAI		
Pramipexole	D2 agonist	0.5 mg, p.o.	[97]	12	VAS	0	
		0.25 mg, p.o.	[108]	10	POMS	↓	0.5 mg ↓ euphoria and energy as measured by ARCI, ↓ POMS
		0.5 mg, p.o.			ARCI		vigor and positive mood and ↓ item “like drug” on DEQ
					DEQ		
		0.5 mg, p.o.	[109]	32	VAS	0	
Tolcapone	COMT	200 mg, p.o.	[110]	25	POMS	0	
	inhibitor	200 mg, p.o.	[111]	23	POMS	0	
		100 mg p.o. day 1	[112]	47	POMS	0	
		followed by 200					
		mg p.o. x 6 days					

For the purpose of this table, measures of positive mood include the ARCI MBG subscale, POMS “Elated” subscale, and the VAS items “High,” “Rush,” “Euphoria,” “Contentedness,” “Like Drug,” and “Good Effects.” Abbreviations: AMS, Adjective Mood Scale. ARCI, Addiction Research Center Inventory. NR, not reported. 0, No change. PANAS, Positive and Negative Affect Scales. POMS, Profile of Mood States. VAS, visual analog scales. STAI, State Trait Anxiety Inventory.

The inability to detect effects of L-DOPA on positive mood does not preclude a relationship between DA neurotransmission, personality traits and goal-directed behavior [11], [19]–[22], [62], [63]; moreover, enhancements in goal-directed behavior may lead to elevated mood [11], [62]–[64]. However, the present results suggest that drug-induced mood-elevating effects are more closely related to neurotransmitters other than DA [3], [37], [62]–[69], perhaps serotonin, norepinephrine, glutamate, GABA, endocannabinoids and endogenous opioids [2], [70]–[75].

If DA's influence on reward seeking behaviors is not accounted for by enhanced pleasure, this raises the question of why it has these effects. Perhaps the best-supported alternative interpretation from the animal literature proposes that DA enhances the incentive salience of reward related cues, increasing their ability to elicit focused interest and sustain effortful seeking [2], [43], [76]. This conclusion is largely based on extensive evidence that decrements in DA neurotransmission reduce the willingness to work for rewards [37], [76] without changing responses in an index of feeding related pleasure [2], [43]. Accumulating work in humans supports this interpretation also ([62], Table 2). For example, in a series of studies conducted here, decreasing DA neurotransmission disrupted the tendency of subjects to respond preferentially to reward-related cues [55] and decreased the willingness to work for abused drugs [53], [58] and monetary reward [Cawley et al, unpublished observations] on progressive ratio breakpoint schedules; each of these effects was produced without reductions in pleasure. Indeed, the majority of studies in humans have failed to replicate an ability of various DA lowering manipulations to diminish drug-induced pleasure [45]–[58].

The present results should be considered in light of the following. First, there was no direct measure of the ability of L-DOPA to increase DA, leaving open the possibility that mood changes were not detected because L-DOPA failed to increase DA levels. However, this seems unlikely since similar doses of L-DOPA given to healthy human volunteers induce behavioural effects [77]–[79] and increase striatal DA synthesis [80]. Pre-clinical studies confirm that L-DOPA increases DA levels in the intact brains of healthy animals, albeit to a lesser extent than in animal models of Parkinson's disease [81]. Although, to our knowledge, there are no reports of L-DOPA induced DA release in healthy humans, the administration of 250 mg more than doubles ventricular CSF levels of the DA metabolite, DOPAC [82]. Moreover, robust L-DOPA induced DA responses have been seen in patients with Parkinson's disease [83]; intriguingly, these effects are largest in those who have developed pathological gambling and the “DA dysregulation syndrome” [84], [85]. Moreover, in these patients, larger L-DOPA-induced DA responses are associated with higher novelty- and fun-seeking personality traits, greater L-DOPA-induced psychomotor activation, and greater drug “wanting” but not drug “liking” [84]. Testing the effect of larger increases in DA neurotransmission in healthy human volunteers will be difficult, though, since higher doses of all currently available drugs that selectively augment DA neurotransmission are limited by side effects such as nausea, vomiting, dizziness and drowsiness. Indeed, this limitation guided our selection of L-DOPA doses in the present study. Second, we used a median split to determine the high and low sub-groups for each of the approach-related personality traits. It might be necessary to recruit participants from the more extreme ends of the normative population distribution for each of these traits in order to detect a differential effect of a DAergic drug, since individual differences in DA neurotransmission might be more pronounced in these more extreme ends of the distribution. This noted, a *post hoc* examination of our more extreme upper and lower quartiles also failed to identify an effect on mood (all p-values≥0.15). Finally, it is possible that an effect on mood would have been seen with a larger sample size. However, this is considered unlikely. The single largest effect size was peak change to ‘Energetic-Tired’ scores (d = 0.339), and this would have required a sample of 138. All other effects would require samples larger than 200. Following corrections for multiple comparisons, these numbers increase further again.

In conclusion, L-DOPA failed to produce changes in positive mood states in a group of healthy human volunteers. These findings add to an accumulating literature suggesting that increases in DA neurotransmission are not sufficient to directly generate positive emotions.

## Methods

### Ethics Statement

The study was carried out in accordance with the Declaration of Helsinki and was approved by the Research Ethics Board of the McGill University Hospital Centre. All subjects gave informed written consent.

### Subjects

Fifty participants were recruited from the McGill University campus through online classified advertisements. Forty-eight men and women (29 females and 19 males; mean age 21.9±3.7 years) completed the study. One participant was excluded due to vomiting at the beginning of the test session and another was excluded because of failure to comprehend the task instructions. All were healthy, as determined by a physical exam, standard laboratory tests, and an interview with the Structured Clinical Interview for DSM-IV, axis I [86]. None had a personal history of axis I psychiatric disorders. On the test day, all subjects tested negative on a urine drug screen sensitive to cocaine, opiates, phencyclidine, barbiturates, Δ^9^-tetrahydrocannabinol, and amphetamines (Triage Panel for Drugs of Abuse, Biosite Diagnostics©, San Diego, CA).

### Procedure

Participants completed the personality questionnaires on the same day as the psychiatric interview, while the test session took place on a separate day. Participants also completed a battery of cognitive tasks during the test session, but these results will be reported elsewhere. Participants were assigned to one of three drug groups (n = 16 per group): placebo, L-DOPA/carbidopa (Sinemet, 100mg/25 mg) or L-DOPA/carbidopa (Sinemet, 200 mg/50 mg), in a randomized, double blind, between-groups design. A combination drug, including the peripheral decarboxylase inhibitor carbidopa, was used to prevent the conversion of L-DOPA to DA before it entered the brain. Low doses of L-DOPA were administered in an effort to avoid the potential confound of side effects such as nausea, vomiting and dizziness. On the test day, participants arrived in the laboratory at 11:30 AM and completed baseline subjective state questionnaires and drug screening. At 12:30 PM, participants ingested two green capsules containing either placebo or one of the two doses of L-DOPA. Participants completed the mood questionnaires at three additional times: 45 minutes, 105 minutes and 165 minutes post-capsule ingestion. Cognitive testing commenced 45 minutes following ingestion of the capsules, coinciding with the time to peak blood concentration of L-DOPA and lasted until 3:30 PM. Female participants who were not taking oral contraceptives were tested within 10 days of the start of menstruation because previous studies have shown that females are more sensitive to reward in the follicular compared to the luteal phase of the menstrual cycle [87]–[89].

### Personality Measures

All subjects completed the Tridimensional Personality Questionnaire (TPQ) [90], Substance Use Risk Profile (SURPS) [91] and the Neuroticism-Extroversion-Openness Five Factor Inventory (NEO-FFI) [92]. Of specific interest in the present study were the TPQ Novelty Seeking factor and two of its subscales (NS1, Exploratory-Excitability and NS2, Impulsiveness), the SURPS factors Impulsivity and Sensation Seeking, and the NEO-FFI factor Extraversion. Each drug group was further sub-divided into high and low groups based on a median split of these personality factor scores for each subject.

### Mood and Subjective Effects Measures

Subjective effects were measured with the bipolar Profile of Mood States (POMS), a sensitive measure of small rapid changes in mood [93], [94], and a visual analog scale (VAS) labeled “Nauseous”. The POMS is comprised of 72 adjectives that describe various mood states. Participants indicate the extent to which they feel these states at each time point on a scale ranging from 0 (“not at all”) to 4 (“extremely”). The POMS items are then converted into 6 empirically derived sub-scales: Elated-Depressed, Composed-Anxious, Agreeable-Hostile, Confident-Unsure, Energetic-Tired and Clearheaded-Confused. Both questionnaires were administered at four times on the test day: at baseline, and at 45, 105 and 165 minutes post-capsule ingestion.

### Data Analyses

Data analyses were conducted using SPSS Statistics (version 18.0; IBM, Chicago, Illinois). Each drug group was further subdivided based on a median split of scores for the approach-related personality traits of Impulsivity, Extraversion, Sensation Seeking and Novelty Seeking, yielding high and low groups for each factor. Three separate analyses were conducted for TPQ Novelty Seeking: the total score as well as scores for the Exploratory-Excitability (NS1) and Impulsiveness (NS2) subscales. Three-way mixed design ANOVAs were used to assess the effects of drug group (independent factor, 3 levels: placebo, 100 mg L-DOPA, 200 mg L-DOPA) and personality trait sub-group (independent factor, 2 levels: high and low) across time (repeated factor, 4 levels: baseline, +45 minutes, +105 minutes and +165 minutes) for all of the mood and subjective effects measures. Two-way independent groups ANOVAs were used to assess the effects of drug group and personality trait subgroup on POMS absolute peak change scores, calculated as the largest difference between any of the three time points and baseline. Post-hoc Least Significant Differences (LSD) tests were used whenever an ANOVA yielded a significant result. The significance for all statistical tests was p<0.05.
